# Anthocyanins and Anthocyanin Biosynthesis Gene Expression in *Passiflora* Flower Corona Filaments

**DOI:** 10.3390/plants14071050

**Published:** 2025-03-28

**Authors:** Eliana Nutricati, Erika Sabella, Carmine Negro, Samar Min Allah, Andrea Luvisi, Luigi De Bellis, Rita Annunziata Accogli

**Affiliations:** Department of Biological and Environmental Sciences and Technologies (DiSTeBA), Salento University, Via Prov. le Lecce-Monteroni, 73100 Lecce, Italy; eliana.nutricati@unisalento.it (E.N.); carmine.negro@unisalento.it (C.N.); samar.minallah@unisalento.it (S.M.A.); andrea.luvisi@unisalento.it (A.L.); rita.accogli@unisalento.it (R.A.A.)

**Keywords:** *Passiflora*, anthocyanin profiles, flower pigmentation, anthocyanin biosynthetic pathway, flavonoids

## Abstract

The diversity in anthocyanin flower pigmentation is vital in the ornamental plant market. To understand the regulation of the corona filament pigmentation of the *Passiflora* flower, we investigated the anthocyanin profiles of five distinct species (*P. violacea*, *P. caerulea*, *P. edulis*, *P. incarnata*, and *P. coccinea*) using HPLC-MS. A total of 14 anthocyanins, differentially distributed in the analyzed species, were identified as responsible for the differences in corona color, which can be attributed to different ratios of pelargonidin, cyanidin, and delphinidin. Additionally, we evaluated the expression of some biosynthetic genes, including *dehydroflavonol reductase* (*DFR), flavonoid 3′-hydroxylase (F3′H)*, and *flavonoid 3′,5′-hydroxylase (F3′5′H)*. *F3′H* seems to regulate the accumulation of cyanidins, *F3′5′H* determines blue pigmentation, and *DFR* enhances the biosynthesis of pelargonidins. Furthermore, three genes coding for key transcription factors, *Myeloblastosis (MYB)*, *basic helix-loop-helix (bHLH)*, and *WD repeat protein (WD40)*, were examined using qPCR. The results confirm that such genes regulate anthocyanin biosynthesis and provide insight into the molecular mechanisms that underlie pigment biosynthesis for application in biotechnologies.

## 1. Introduction

Flower pigmentation is an important biological trait because it attracts pollinators, aids reproduction, and provides resilience to environmental challenges; in addition, it represents an ornamental feature with significant economic value. The *Passiflora* genus, also known as passion flowers or passion vines, represents the largest genus of the Passifloraceae family with about 600 species [1,2], and it is characterized by flowers of diverse colors and morphological features [3,4]. A peculiar trait of Passiflora flowers is the corona, a filamentous organ between the petals and stamen whorls. This organ, important for pollination, is characterized in naturally occurring species by a high variability in size and morphology, and in a range of color combinations in many species [3].

Flower pigmentation primarily arises from biochemical processes, including the synthesis of three major classes of pigments: anthocyanins [5], carotenoids, and betalains [6]. The concentration of these pigments in the flowers determines their color intensity; therefore, flowers with greater coloration are associated with higher concentrations of specific pigments. Among flavonoids, anthocyanins are the main pigments that cause a broad variety of colors, ranging from orange to blue, in many flowers, fruits, and vegetables [7,8]. The different flower colors are mainly due to the chemical structure of the different anthocyanins or anthocyanidins that are synthesized in the flower [9]. Anthocyanins are common water-soluble pigments which are classified into three main types depending from which anthocyanidin they derive from: pelargonidin (Pg), cyanidin (Cy), and delphinidin (Dp) [10]; methylation by methyltransferase (MTase) of cyanidin results in peonidin, while mono- and bimethylation of delphinidin give petunidin and malvidin, respectively [11].

Anthocyanins have high potential as dye and food colorants due to their several colors, ranging from orange, red, blue, and purple, as well as their water solubility, allowing their incorporation into aqueous food systems [12]. In addition, anthocyanins have been traditionally used as a medicine against various diseases, mainly due to their health-promoting antioxidants and antimicrobial effects [13,14]. Anthocyanins found in fruits, vegetables, and flowers have protective effects against cardiovascular diseases and cancer [15,16,17].

Previous studies on *Passiflora* have shown that the anthocyanins present are responsible for floral pigmentation, whilst other pigments, as carotenoids and betalains, have not been reported. Aizza et al. [18] identified several different anthocyanins, such as cyanidin, pelargonidin, petunidin, peonidin, and malvidin, in the corona filaments of different species of *Passiflora* (*P. incarnata*, *P. coccinea*, and the hybrid Lady Margaret). Among *Passiflora* species, some have edible flowers; Teixeira et al. [19] included *P. incarnata* in the list of anthocyanin-rich edible flowers, which represent an innovative type of vegetal food due their potential health benefits.

Usually, in order to measure anthocyanin composition in different parts of a plant, HPLC/MS and/or NMR are utilized. However, in recent years, many innovative techniques such as multiomics, a widely targeted metabolite modificomic strategy, have been developed to efficiently identify metabolite modifications in plants [20].

**Figure 1:**
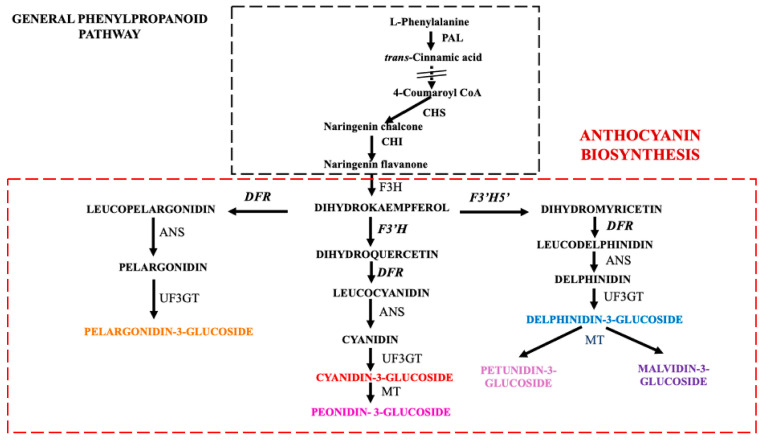
The anthocyanin biosynthesis pathway. Genes analyzed by qPCR are reported bold, while the name of different anthocyanin’s types are shown in different colors. PAL, phenylalanine ammonia lyase; CHS, chalcone synthase; CHI, chalcone isomerase; F3H, flavanone 3-hydroxylase; F3′H, flavonoid 3′-hydroxylase; F3′5′H, flavonoid 3′5′-hydroxylase; DFR, dehydroflavonol reductase; ANS, anthocyanidin synthase; UF3GT, UDP-glucose: flavonoid 3-O-glucosyltransferase; MT, methyltransferase.

In plants, the accumulation of anthocyanins occurs through a distinct pathway, involving different enzymes, encoded by respective genes that have been widely studied [21,22,23]. The first step of the anthocyanin biosynthesis pathway (Figure 1) is catalyzed by chalcone synthase (CS), followed by chalcone isomerase (CHI) and flavanone hydroxylase (F3H), producing dihydrokaempferol. Three different pathways depart from this compound. Flavonoid 3′-hydroxylase (F3′H) produces the substrate (dehydroquercetin) of dihydroflavanol 4-reductase (DFR), which catalyzes the production of leucocyanidin, addressing the subsequent production of cyanidin-3-glucoside and peonidin-3-glucoside. Instead, flavonoid 3′, 5′-hydroxylase (F3′5′H), starting from dihydrokaempferol, leads towards the synthesis of delphinidin-3-glucoside, petunidin-3-glucoside, and malvidin-3-glucoside. The third pathway is also regulated by DFR, which, from dihydrokaempferol, conduces the formation of pelargonidin-3-glucoside. The role of DFR, F3′H, and F3′5′H is to define the type of anthocyanin accumulated [23], as shown in Figure 1, where the anthocyanin biosynthetic pathway, a part of the general phenylpropanoid pathway, is schematized. Colors ranging from red to purple and blue are produced by different patterns of hydroxylation, methylation, glycosylation, and possible acylation of the anthocyanidins [18].

A second group of genes required for anthocyanin biosynthesis includes genes coding for transcription factors, which regulate the expression of structural genes; among them are myeloblastosis (*MYB*), basic helix–loop–helix (*bHLH*), and WD40-repeat protein (*WD40*), which are the most-studied. It is known that MYB transcription factor, through interaction with bHLH and WD40, constitutes a regulatory complex involving anthocyanin accumulation [24,25]. The *bHLH* genes are involved in the response to light [26], hormone signals [27], and seed germination [28], and in the regulation of anthocyanin biosynthesis [29]. Transcription factors (TFs) have key roles in regulating growth and responding to stress in plants [30]. Among TF genes, *bHLH* and *MYB* are widely identified in all plant genomes [31]. Furthermore, in Arabidopsis, *AtCIB1* and *AtCRY* control the expression of flowering genes [32], in pepper, *CabHLH33* is overexpressed in flower buds [33], and a homolog gene, *AtbHLH31*, induces petal growth, regulating cell expansion [34].

Although many studies about anthocyanins have been carried out in different species, data regarding anthocyanin accumulation in *Passiflora* are limited to *P. suberosa*, *P. edulis*, and *P. incarnata* [18,35]. Due to the limited sequence availability of species deposited in public databases, a genomic approach to study anthocyanin synthesis was employed by Aizza et al. [36] in the PASSIOMA project which individuated 15 different genes of the anthocyanin pathway in *P. edulis.* In recent years, Xu et al. [37] have identified key genes involved in flavonoid biosynthesis in *P. edulis*.

To date, no research has been conducted on the expression and regulation of anthocyanin biosynthesis genes in *Passiflora* flowers; however, recently, some authors [38,39,40] have reported integrated metabolomic, proteomic and genomic analyses focused on anthocyanin biosynthesis in passion fruit pericarp, indicating the importance of several key metabolites and genes, mainly of the general phenylpropanoid pathway (Figure 1).

We chose five different species (including the hybrid *P. violacea*) with different colors and morphologies of floral corona to investigate, for the first time, the mechanisms underlying the corona color variability of *Passiflora* flowers through combined metabolic and gene expression approaches. For this purpose, we selected three genes (*DFR*, *F3‘H*, and *F3′5′H*) that, starting from dihydrokaempferol, regulate the supply of substrates to the three metabolic pathways that lead to pelargonidin-3-glucoside, cyanidin-3-glucoside/peonidin-3-glucoside, and delphinidin-3-glucoside/petunidin-3-glucoside/malvidin-3-glucoside, respectively (Figure 1). In addition, we opted to analyze one gene from each transcription factor family (*MYB*, *bHLH*, and *WD40*) that regulates the expression of anthocyanin biosynthesis genes.

The findings will serve as a foundation for future developments in the usage of *Passiflora* as the food industry works to improve product safety and quality in order to meet the needs of consumers by decreasing the use of artificial additives and instead utilising natural products.

## 2. Results

### 2.1. Identification and Quantification of Anthocyanins in Different Passiflora Species

**Figure 2:**
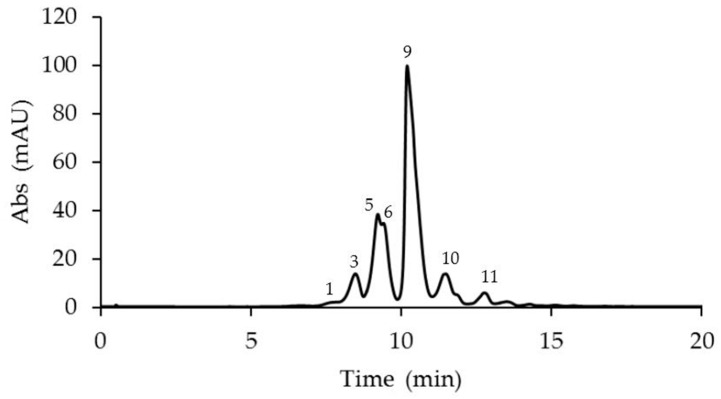
A representative chromatogram recorded at 520 nm of *Passiflora violacea* extract for the identification of anthocyanins. Peak 1: cyanidin 3-O-sophoroside; 3: delphinidin 3-O-glucoside; 5: cyanidin 3-O-glucoside; 6: cyanidin 3,5-O-diglucoside; 9: cyanidin 3-O-rutinoside; 10: pelargonidin 3,5-O-diglucoside; and 11: peonidin 3,5-O-diglucoside.

**Figure 3:**
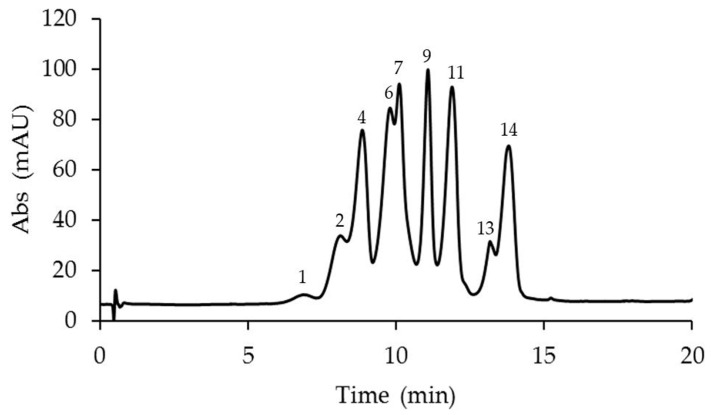
A representative chromatogram recorded at 520 nm of *Passiflora caerulea* extract for the identification of anthocyanins. Peak 1: cyanidin 3-O-sophoroside; 2: petunidin 3-O-rutinoside; 4: petunidin 3,5-O-diglucoside; 6: cyanidin 3,5-O-diglucoside; 7: malvidin 3,5-O-diglucoside; 9: cyanidin 3-O-rutinoside; 11: peonidin 3,5-O-diglucoside; 13: peonidin 3-O-rutinoside; and 14: malvidin 3-O-rutinoside.

**Figure 4:**
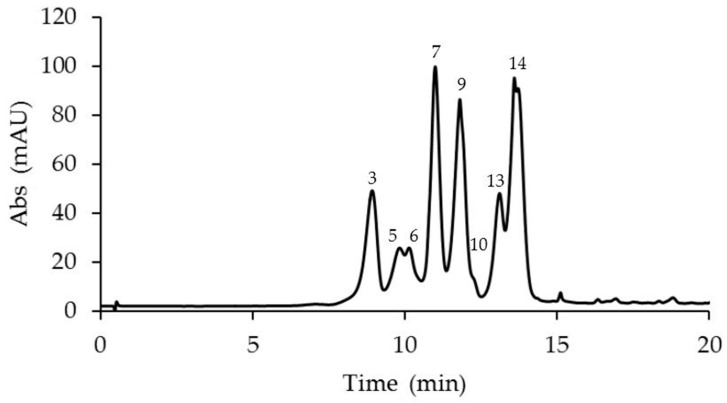
A representative chromatogram recorded at 520 nm of *Passiflora edulis* L. extract for the identification of anthocyanins. Peak 3: delphinidin 3-O-glucoside; 5: cyanidin 3-O-glucoside; 6: cyanidin 3,5-O-diglucoside; 7: malvidin 3,5-O-diglucoside; 9: cyanidin 3-O-rutinoside; 10: pelargonidin 3,5-O-diglucoside; 13: peonidin 3-O-rutinoside; and 14: malvidin 3-O-rutinoside.

**Figure 5:**
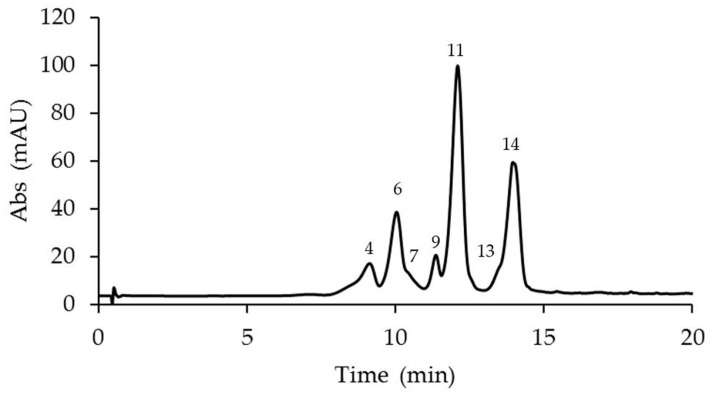
A representative chromatogram recorded at 520 nm of *Passiflora incarnata* extract for the identification of anthocyanins. Peak 4: petunidin 3,5-O-diglucoside; 6: cyanidin 3,5-O-diglucoside; 7: malvidin 3,5-O-diglucoside; 9: cyanidin 3-O-rutinoside; 11: peonidin 3,5-O-diglucoside; and 13: peonidin 3-O-rutinoside; 14: malvidin 3-O-rutinoside.

**Figure 6:**
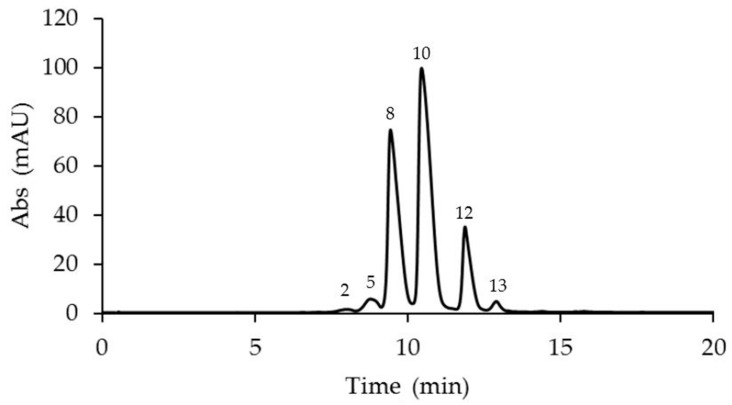
A representative chromatogram recorded at 520 nm of *Passiflora coccinea* extract for the identification of anthocyanins. Peak 2: petunidin 3-O-rutinoside; 5: cyanidin 3-O-glucoside; 8: pelargonidin 3-O-glucoside; 10: pelargonidin 3,5-O-diglucoside; 12: pelargonidin 3-O-rutinoside; and 13: peonidin 3-O-rutinoside.

The anthocyanin analyses revealed the presence of 14 anthocyanins identified in the corona filaments of five mature *Passiflora* flowers (Figure 2, Figure 3, Figure 4, Figure 5 and Figure 6, Table 1). Anthocyanin identification was carried out through both comparison with authentic analytical standards (cyanidin 3-O-glucoside and cyanidin 3,5-O-glucoside) and the literature data [18,41,42]. Figure 2, Figure 3, Figure 4, Figure 5 and Figure 6 show the main anthocyanin peaks after the HPLC of the different samples, indicating each peak with a number in relation to the retention time on the chromatographic column, as shown in Table 1.

Chromatographic runs revealed the presence of a minimum of six different anthocyanins in the corona filaments of *P. coccinea* and up to nine anthocyanins in *P. caerulea*, in the corona’s filaments of which an intricate combination of anthocyanins is reported. Six major anthocyanidins, pelargonidin, cyanidin, malvidin, peonidin, delphinidin, and petunidin, were identified in the analyzed samples. Most of the corresponding anthocyanins were present as monoglucosides or diglucosides.

*P. violacea* corona filament extract was characterized by the highest content of delphinidin 3-O-glucoside and by four cyanidin-based anthocyanins (Figure 2, Table 1). In addition, the relevant presence of pelargonidin 3,5-O-diglucoside and peonidin 3,5-O-diglucoside confers a coloration ranging from bright red to intense purple. *P. caerulea* is characterized by the greatest variability in the presence of anthocyanins, while pelargonidin and delphinidin-based anthocyanins were not detected (Figure 3, Table 1). Instead, in *P. edulis* corona filaments (Figure 4, Table 1) malvidin-based anthocyanins were especially abundant as malvidin 3-O-rutinoside (135.72 mg/g FW) and malvidin 3,5-O-diglucoside (117.11 mg/g FW). The anthocyanin composition of *P. incarnata* corona filaments (Figure 5, Table 1) was characterized by the highest level of peonidin 3,5-O-diglucoside (110 mg/g FW); malvidin 3-O-rutinoside was the second most abundant anthocyanin after monoglucoside. In *P. coccinea*, we found an abundance of pelargonidins which are responsible for the deep red color of the corona filaments (Figure 6, Table 1); they were present as pelargonidin 3-O-glucoside, pelargonidin 3-O-rutinoside, and pelargonidin 3,5-O-diglucoside, the most abundant (778.84 mg/g FW), and also among the five *Passiflora* samples analyzed. Further, petunidin 3-O-rutinoside, cyanidin 3-O-glucoside, and peonidin 3-O-rutinoside were detected.

### 2.2. Expression Profiles of the Anthocyanin Biosynthesis-Related Genes

**Figure 7:**
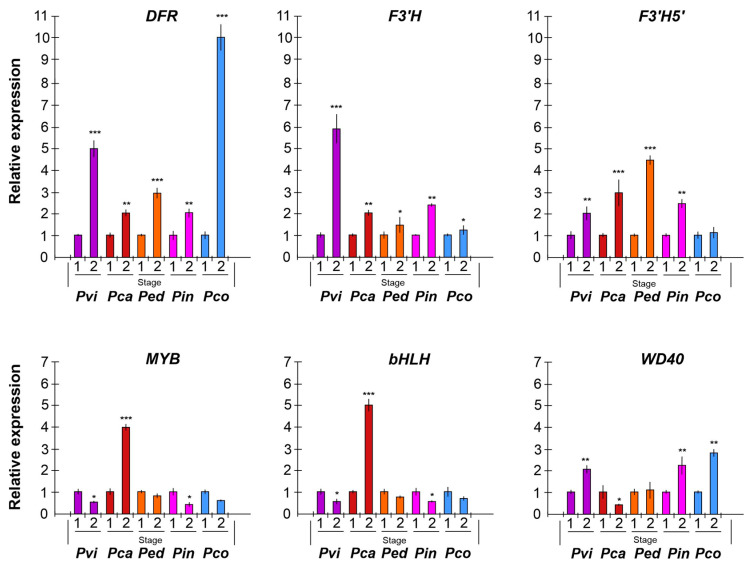
Expression analysis of anthocyanin biosynthesis genes *DFR* (dehydroflavonol 4-reductase), *F3′H* (flavonoid 3′-hydroxylase), *F3′5′H* (flavonoid 3′,5′-hydroxylase); gene coding transcription factors, *MYB* (myelobastosis), *bHLH* (basic helix-loop-helix), and *WD40* (WD repeats protein). The *EF1a* (elongation factor) gene was used as the reference gene. The analysis was carried out on corona filaments at stage 1 (floral bud) and stage 2 (mature flower). *Pvi: Passiflora violacea* (purple); *Pca: Passiflora caerulea* (red brown); *Ped: Passiflora edulis* (orange); *Pin: Passiflora incarnata* (dark pink); *Pco: Passiflora coccinea* (light blue). ANOVA results were reported based on their statistical significance. * *p* < 0.05, ** *p* < 0.01, *** *p* < 0.001.

To determine the molecular mechanism regulating corona filament pigmentation in *Passiflora*, an expression analysis was performed through qRT-PCR on some key genes for anthocyanin biosynthesis (Figure 7). Two stages of development were considered: a floral bud (stage 1) and a full-blooming (mature) flower (stage 2).

In the corona filaments, the *DFR* gene was highly expressed in mature flowers compared to floral buds, with significant up-regulation in *P. coccinea* (a 10-fold change), which is characterized by deep red corona filaments, and in *P. violacea* (a 5-fold change), with corona filaments of a reddish-purple color. *F3′H* showed high expression in *P. violacea* in stage 2; the transcript was also up-regulated in *P. incarnata* flowers compared to buds. In *P. caerulea*, *P. edulis*, and *P. coccinea*, the gene did not show a significant change in gene expression. On the contrary, *F3′5′H* expression increased at flower maturity in *P. edulis* (about a 4.5-fold change), *P. caerulea* (a 3-fold change), and *P. incarnata* (a 2.5-fold change), while it did not evidence any significant variation of the expression in *P. coccinea*.

Regarding the expression analysis of transcription factors, an interesting finding is that *bHLH* and *MYB* genes showed similar trends. Both genes were down-regulated in all samples, except for *P. caerulea*, where the level of transcripts increased at stage 2 with a 4- and 5-fold change. In contrast, the gene coding for WD40 showed the opposite trend compared with *bHLH* and *MYB*: in *P. violacea*, *P. incarnata*, *P. coccinea*, and *P. edulis* the gene was up-regulated in mature flowers, whilst in *P. caerulea* the gene was down-regulated.

## 3. Discussion

### 3.1. Anthocyanin Presence in Corona Filaments

Anthocyanins are essential pigments that determine flower colors; generally, cyanidin, peonidin, and pelargonidin contribute to the red color, while delphinidin, petunidin, and malvidin contribute to the blue color [43,44]. Although the final pigmentation depends on several factors, such as the basic structure, co-pigmentation, and vacuolar pH, the anthocyanin combination is the basic element in determining flower pigmentation.

**Figure 8:**
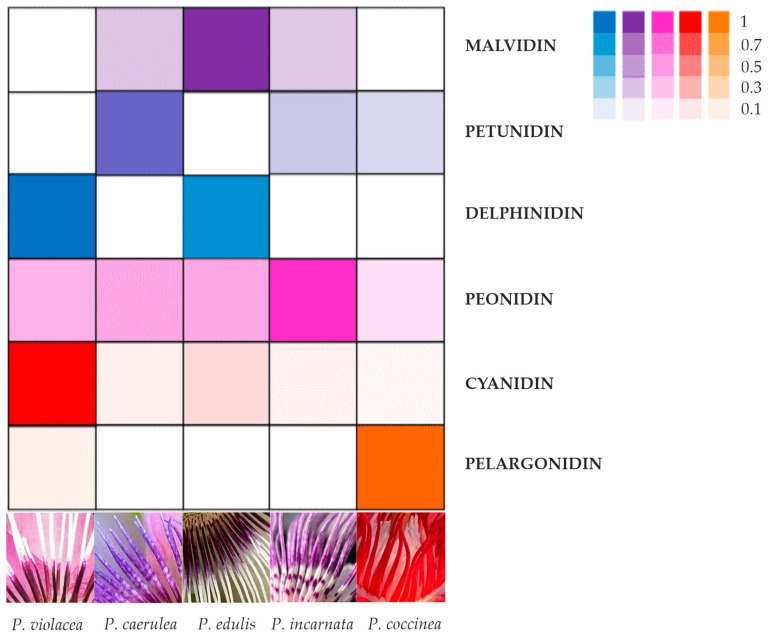
Imaging showing the main anthocyanins (cyanidin, pelargonidin, and delphinidin) and their derivative (peonidin, petunidin, and malvidin) distribution among five *Passiflora* species (*P. coccinea*, *P. violacea*, *P. caerulea*, *P. edulis*, and *P. incarnata*). The color intensity in the boxes is proportional to the amount of anthocyanin detected in the corona filaments of each *Passiflora*, while the white boxes indicate that the corresponding anthocyanin was undetected.

This study focused on the evaluation of the anthocyanin content in the corona filaments of five *Passiflora* species. We chose species of *Passiflora* characterized by extreme differences in the color of their corona filaments. The HPLC/ESI/TOF data allowed for the identification of 14 anthocyanins, which were distributed in various amounts and in different combinations, giving the high variability observed in corona filaments. The results obtained can be summarized with an image, evidencing the relative anthocyanin distribution and the base color of the corona filaments for each *Passiflora* species (Figure 8).

Delphinidin is the main anthocyanin responsible for the deep blue to purple color in flowers [45]. We found that blue–purple color of the corona mostly accumulated due to delphinidin, as was the case for *P. edulis*, but corona filaments with reddish purple color accumulated mainly due to delphinidin and cyanidin, as shown for *P. violacea*, which is characterized by reddish purple filaments at the base.

Therefore, from the observation of corona filament pigmentation, we divided the five samples into four groups: deep red including *P. coccinea*, deep purple (*P. violacea*), violet-blue (*P. edulis*), and lilac or pale purple (*P. caerulea* and *P. incarnata*). The anthocyanin composition differed strongly among these groups: *P. coccinea* represents the only species among those analyzed with a great content of pelargonidin, that, in combination with petunidin and peonidin, cause an intense red color in corona filaments. The deep purple coloration of the *P. violacea* corona is unique to this species and results from a special combination of the highest concentration of cyanidins (which are responsible for the purple pigmentation) and the highest quantity of delphinidin. *P. edulis* and *P. incarnata* are characterized by a corona with different hues of purple and blue; the blue of the tip filaments is due to the presence of delphinidin, the deep purple base is due to malvidin, and *P. incarnata* corona, with a greater portion of light purple, could be explained by the presence of peonidin, a methylated derivative of cyanidin. The results are in accordance with Mori et al. [46], who found that blue flowering grape hyacinth accumulated delphinidin, and that lilac or reddish-purple colors are determined by the different combinations of delphinidin and cyanidin. In *P. caerulea*, the methylation of the main anthocyanidins, cyanidin (modified in peonidin) and delphinidin (converted in petunidin and/or malvidin), gives a pigmentation light purple with pink sections to corona filaments. In a study carried out on hyacinth flowers, Lou et al. [42] underlined that petunidin 3-glucoside and malvidin 3-glucoside are responsible for violet blue or purple coloration in grape hyacinth. In our investigation, the fading of the color into pink in the spot of filaments could be attributed to the presence of peonidin. In peony, the methylation of the anthocyanidin conferred a purple pigmentation [47] in grape (*Vitis vinifera*), and malvidin 3-glucoside and peonidin 3-glucoside resulted in red-skinned grapes [48].

These observations suggest that anthocyanin modification plays a key role in the final coloration of flowers of *Passiflora* according to results reported in the previous literature regarding different plant species [42].

### 3.2. Expression Analysis of Anthocyanin Pathway Genes and Transcription Factors

The expression of *DFR*, *F3′H*, and *F3′5′H* correlates almost perfectly with the anthocyanin presence in each *Passiflora* sample. In fact, in the anthocyanin biosynthesis pathway, DFR catalyzes the conversion of the key metabolite dihydrokaempferol to leucopelargonidin, a precursor for pelargonidin synthesis. Also, the enzyme DFR acts alongside the other two branches of anthocyanin biosynthesis (leading to cyanidin/peonidin 3-glucoside and delphinidin/petunidin/malvidin 3-glucoside, respectively) downstream of *F3′H* and *F3′5′H*. This means that a high expression of *DFR* can lead directly to the production of pelargonidin 3-glucoside and, with a simultaneous high expression of *F3′H* and/or *F3′5′H*, to the production of high amounts of cyanidin/peonidin 3-glucoside and delphinidin/petunidin/malvidin 3-glucoside, respectively (Figure 1).

The hydroxylation on the B ring of anthocyanidins is determined by flavonoid 3-hyhdroxylase (F3′H) and flavonoid 3′5′-hydroxylase (F3′5′H), which belong to the P450 (CYP75) protein family. Both enzymes catalyze the hydroxylation of the flavonoid B-ring but in a different position, at the 3′- or the 3′- and 5′-position, leading to red/pink or violet/blue colored anthocyanins, respectively.

The expression analysis showed in Figure 7 indicates that in *P. coccinea*, with red-colored corona filaments, *DFR* expression was higher when the flower was at full bloom (stage 2) compared to the floral bud. On the other hand, *F3′H* and *F3′5′H* transcripts remain stable, suggesting that the alternative pathway for cyanidins and delphinidins is not particularly active. The data are, therefore, in accordance with the highest content of pelargonidins and low levels of cyanidins and delphinidins in the corona filament of *P. coccinea*.

In *P. violacea DFR*, *F3′H* and *F3′5′H* were up-regulated at stage 2, in line with the results regarding the anthocyanin mix in corona, characterized by a high level of cyanidins, a consistent amount of pelargonidin, and the presence of delphinidin 3-O-glucoside (Figure 8, Table 1). *P. caerulea* includes the greatest number of anthocyanin forms, except for pelargonidins, as evidenced by the expression trend of *DFR*, *F3′H*, and *F3′5′H*: the three transcripts increased during flower maturation, with a greater expression of *F3′5′H* (about a 3-fold change), determining the synthesis of more delphinidin derivatives (Figure 8, Table 1). The combination of different anthocyanins determines the pink–purple color of corona filaments.

Although *P. incarnata* and *P. edulis* corona seem to have a similar color, the differences in terms of anthocyanin content are confirmed by the similar increase in the expression of all three genes (Figure 8).

Our results confirm that structural genes, such as *F3′H*, *F3′5′H*, and *DFR*, regulate different biosynthetic branches of the anthocyanin pathway. In fact, different ornamental plants have been genetically engineered through the manipulation of such genes to change the anthocyanin composition and, in turn, modify the flower color [6]. *Petunia* was the first ornamental plant that was modified through the overexpression of a gene coding DFR, resulting in am orange flower with a high content of pelargonidins [49].

In a review, Mekapogu et al. [50] showed that the floral color of many plant species has been modified by the overexpression, downregulation, and silencing of a specific anthocyanin key biosynthetic gene. For example, in cyclamen, Boase et al. [51] suppressed the *F3′5′H* gene to obtain a shift in flower pigmentation from purple to red/pink, whereas the delphinidin pathway was enhanced by expressing a chimeric pansy *F3′5′H*, resulting in violet/blue chrysanthemum flowers [52]. In *Gerbera*, the overexpression of the *DFR* gene resulted in a shift in the anthocyanin pathway from delphinidin to pelargonidin [53].

In tobacco, Nakatsuka et al. [54] observed that the expression of the *Gentiana GtF3′H* gene resulted in an increase in anthocyanin content and flower color intensity. Moreover, the down-regulation of *F3′H* and *F3′5′H* in *Torenia* hybrida, which accumulates delphinidin and cyanidin, produced a pale, pink-colored flower with mostly pelargonidin [55]. In line with these findings, Gopaulchan et al. [56] suggested that *F3′H* expression may be involved in determining the shade color intensity in red and pink spathes of *Anthurium*. The increased expression of *F3′H* and *F3′5′H* in *Petunia* resulted in increased anthocyanin production, altering the flower color from pale pink to dark pink [57].

In addition, we considered it important to investigate the role of transcription factors, such as a ternary complex MYB-bHLH-WD40 (MBW), which are known to regulate anthocyanin biosynthesis at the transcriptional level [58]. The results show that *bHLH* and *MYB* cooperate to regulate structural genes for anthocyanin biosynthesis. In fact, in all species of *Passiflora* analyzed, the expression of both genes follows a similar trend: they show a greater transcript level in immature flowers, probably in order to induce the expression of key genes involved in biosynthesis, which in turn reaches the highest level of expression in mature flowers. The exception is represented by *P. caerulea*, showing an increase in *MYB* and *bHLH* expression in flowers at full bloom, probably due to the activation of the three branches of the anthocyanin pathway. The third component of regulation complex, *WD40*, shows an opposite trend as the gene was up-regulated in mature flowers, suggesting its role in stabilizing the complex. Overall, the three TFs induce the expression of the structural genes analyzed for pigmentation in *Passiflora* species.

Previous studies reported that *AcMYB110* plays a key role in determining the red petal color of kiwi flowers [59]. Moreover, there was no activation of *F3′5′H* promoter, even in the presence of the MBW complex in *Actinidia*, suggesting that other regulatory proteins are responsible for the expression of *F3′H* and *F3′5′H* genes [60]. In addition, in *Ipomea purpurea*, the complexes WDR, MYB, and bHLH bind recognition-specific elements of the anthocyanin biosynthetic genes and control the pathway [61]. Finally, we need to underline that the application of the targeted metabolite modificomics strategy of Yang et al. [20] could be useful to provide new relevant information about the mechanism of anthocyanin regulation.

## 4. Materials and Methods

### 4.1. Plant Materials

Passiflora is known to be highly sensitive to cold and thermal excursions; for this reason, individuals from different species were transplanted in pots and moved to the brightest area of the unheated greenhouse of the Botanical Garden of the University of Salento. The growing substrate was a highly potent drainage mixture, composed of universal soil, non-calcareous agricultural soil, and mature compost (1:1:1 ratio); 40 cm diameter, 35 cm height (volume 35 L) earthen pots were employed. To ensure correct drainage, the base of the plot was filled with a 10–15 cm layer of gravel. Irrigation was manually provided when necessary, depending on the species. The first fertilization was conducted during the vegetative stage, while the second one was conducted at the pre-flowering stage using 20 g of multipurpose slow-release fertilizer (Osmocote^®^ Universale, NPK MgO 17-09-11+2, Savina Orazio Innovazioni Tecnologiche per l’Agricoltura, Leverano, Lecce, Italy) per plant.

For each species, three replicas of the corona filaments were harvested at the floral bud stage and at the full blooming stage, weighted (about 250 mg), homogenized by mortar and pestle in liquid nitrogen, and then stored at −80 °C until use.

### 4.2. HPLC/DAD/TOF Analysis

From each species, three replicas of 250 mg FW of corona filaments, reduced to a powder, were extracted with 2.5 mL of CH_3_OH: H_2_O: HCCOH 40:58:2 V/V/V for 30′; the extract was then purified by SPE JTBaker C18 polar plus columns, previously activated with 2 mL of MetOH and 5 mL H_2_O. After the loading of the sample, 2 mL of ethyl acetate were utilized for removing phenolic compounds, and the sample was eluted with MetOH acidified with 2% HCOOH. After the evaporation of the solvent, the cyanidins were solubilized in H_2_O with 2% HCOOH.

The anthocyanins were identified by adhering to the methodology used by Blando et al. [62] with an Agilent series 1200 chromatographic system (Agilent Technologies, Palo Alto, CA, USA) equipped with the Agilent TOF 6230 trough ESI interface in the positive mode. Phase A was water plus 2% of formic acid, and phase B was acetonitrile:water: formic acid 53:45:2. The HPLC column was an Agilent Extended C18 (1.8 µm, 2.1 × 50 mm). Separation was carried out at 40 °C with a gradient elution program at a 0.3 mL/min flow rate. The following multistep linear gradient was applied: 0 min, 0% B; 15 min, 25% B; 30 min, and 50% B. The injection volume of the HPLC system was 10 µL. TOF operated with positive ionization using the internal reference masses of *m*/*z* 121.0508, 149.0233, 322.0481, and 922.0097. Finally, the wavelength of DAD detection was 520 nm. The mass spectrometer conditions were as follows: capillary voltage 3.5 kV in the positive mode; nitrogen was used as the nebulizer and desolvation gas; drying gas temperature: 300 °C; drying gas flow: 12 L/min, and nebulizing gas pressure: 40 psig; finally, the source temperature was 120 °C. The Mass Hunter software (Agilent Technologies, Palo Alto, CA, USA) was used to process the mass data of the molecular ions.

The anthocyanin content was determined using two different standards: cyanidin 3-glucoside for the monoglucoside form and cyanidin-3,5-O-diglucoside for the diglucoside form (Extrasynthese, Genay, France). Calibration curves, the linear concentration range (from 1 to 80 μg/mL), the limit of detection (S/N = 3), the limit of quantification (S/N = 10), and intra/inter-day precision (n = 5) are summarized in Table 2.

### 4.3. Identification of Passiflora Anthocyanin Biosynthesis Genes

To search for the cDNA coding for the anthocyanin biosynthesis enzyme (F3′H), degenerate primers were designed based on the conservative motifs of cDNA from different plant species. The amplification products were first sequenced (Eurofins Genomics, Ebesberg, Germany) to verify the sequence consistency.

The sequences of *DFR*, *MYB*, and *WD40* were derived from cDNA libraries of PASSIOMA Project [36], and the sequence coding for bHLH was derived from a study by Liang et al. [63] on *Passiflora edulis* in response to abiotic stress: *PebHLH126* was highly expressed in corona filaments; the sequence of *F3′5′H* was obtained from data by Xu et al. [37] in the identification of key genes involved in flavonoid biosynthesis in *P. edulis*.

The *F3′H* cDNA was obtained in a present study through RT-PCR using degenerate primers. To confirm that the cDNA obtained coding for a flavonoid 3′-hydroxylase, the partial cDNA obtained was sequenced and compared with other plant sequences using BLAST (blast.ncbi.nlm.nih.gov, version 2.16.0, accessed on 15 January 2025). The results reported in Figure S1 show that F3′H belongs to the CYP75B subfamily, a flavonoid 3-hyhdroxylase.

For each gene, a pair of primers was tested to identify those that gave consistent amplification across all species analyzed.

### 4.4. RNA Extraction and Gene Expression Analysis by qRT-PCR

The total RNA was isolated from 100 mg (FW) of corona filaments (three replicas), previously powdered, using TRIZOL (Invitrogen, Carlsbad, CA, USA). cDNA synthesis was carried out using TaqMan^®^ Reverse Transcription Reagents (Applied Biosystems, Foster City, CA, USA) according to the manufacturer’s protocol. The amplification reactions were performed using the Applied Biosystems^®^ QuantStudio^®^ 3 Real-Time PCR System. Each reaction consisted of 2 ng of cDNA, 12.5 μL of the Power SYBR Green RT-PCR Maste\r mix (Applied Biosystems), 5.0 M-6 forward and reverse primers, and ultrapure DNase/RNase-free water (Carlo Erba Reagents, Cornaredo, Milano, Italy) in a total volume of 25 μL. The cycling conditions were as follows: 2 min at 50 °C and 10 min at 95 °C, followed by 45 cycles of 95 °C for 15 s and 60 °C for 1 min. Melting curve analysis was performed after PCR to evaluate the presence of non-specific PCR products and primer dimers.

The primers (Supplementary Table S1) were designed with the Primer Express Software 3.0 on the mRNA sequences obtained from the literature and from cDNA obtained in this work (*F3′H*). For each sample, the expression of each gene in the anthocyanin biosynthesis pathway was analyzed with three biological replicates of the floral at bud stage and mature flowers, with two technical replicates of each.

Different primer pair combinations for each gene were tested to identify those that gave amplification across all species. Quantitative real-time PCR was used for the rapid and reliable quantification of mRNA transcription. However, selecting an appropriate reference gene was crucial for an exact comparison of the mRNA transcription in different samples. Of the various genes reported in the literature, we employed *EF1a* (elongation factor) as a reference gene, as reported by [63].

For relative quantification of gene expression, we calculated the fold changes (FC) using the following formula:

F = 2^(−∆∆CT)^

where

∆∆CT = [(CT target gene) − (CT reference gene)] mature flower − [(CT target gene) − (CT reference gene)] flower bud.

### 4.5. Statistical Analysis

Data regarding the anthocyanin content were reported as the mean ± SD. Statistical evaluation was conducted using Duncan’s multicomponent test (*p* < 0.05) to discriminate among the mean values. A one-way ANOVA test was applied to the expression gene data.

## 5. Conclusions

The ornamental value of a plant is derived from different aesthetic features, such as the brilliant colors and shapes of the flowers, fruits, and leaves, and the floral aroma. These attributes often have medicinal and nutritional value in some ornamental plants. Floral pigmentation represents the most attractive and beautiful trait of ornamental plants, which also has commercial importance. Moreover, petal color is fundamental for pollinator attraction.

*Passiflora*, in addition to its edible value, has unique medicinal value due to the presence of flavonoids, and has been grown as an ornamental plant for its differences in morphology, petal colors, and tones of flowers. In this work, we analyzed, for the first time, the molecular mechanism of *Passiflora* flower corona filament pigmentation through a comparative metabolomic and molecular analysis of five different *Passiflora* species.

The results show that different combinations and contents of anthocyanins (or anthocyanidins: cyanidin, delphinidin, peonidin, malvidin, pelargonidin, and petunidin) cause peculiar pigmentation typical of each *Passiflora* species. Such results agree with the expression data of some anthocyanin biosynthetic genes (*F3′H*, *F3′5′H*, and *DFR*) which are responsible for directing metabolites to the different branches of the anthocyanin pathway. So, this work provides new insights into the molecular mechanism of flower color in *Passiflora* species, representing a starting point for future applications in plant biology and agriculture.

## Figures and Tables

**Table 1:** Anthocyanin compounds identified in the corona filaments of six different *Passiflora* species by High-Performance Liquid Chromatography coupled to Electrospray Ionization Time-of-Flight Mass Spectrometry (HPLC ESI/MS-TOF) following the extraction from corona flowers of five *Passiflora* species at full blooming.

N.	Compound	M-H+ (Exp.)	M-H+ (Calc.)	Error Δ ppm	Anthocyanins Amount in *Passiflora* Species (μg/g Fresh Weight) **				
					*P. violacea*	*P. caerulea*	*P. edulis*	*P. incarnata*	*P. coccinea*
1	Cyanidin 3-O-sophoroside	611.1612	611.1607	−0.60	20.76 ± 0.61 ^a^	2.54 ± 0.03 ^b^	<LOD	<LOD	<LOD
2	Petunidin 3-O-rutinoside	625.1746	625.1763	2.77	<LOD	15.28 ± 0.21 ^a^	<LOD	<LOD	4.42 ± 0.34 ^b^
3	Delphinidin 3-O-glucoside	465.1030	465.1028	−0.57	90.24 ± 0.03 ^a^	<LOD	71.02 ± 0.25 ^b^	<LOD	<LOD
4	Petunidin 3,5-O-diglucoside	641.1717	641.1712	−0.13	<LOD	43.07 ± 0.25 ^a^	<LOD	21.49 ± 0.36 ^b^	<LOD
5	* Cyanidin 3-O-glucoside	449.1088	449.1078	−2.17	168.5 ± 0.52 ^a^	<LOD	28.17 ± 0.99 ^b^	<LOD	29.13 ± 0.36 ^b^
6	* Cyanidin 3,5-O-diglucoside	611.1610	611.1607	−0.51	176.15 ± 0.38 ^a^	37.78 ± 0.86 ^b^	29.56 ± 0.92 ^b^	38.46 ± 0.21 ^b^	<LOD
7	Malvidin 3,5-O-diglucoside	655.1867	655.1869	0.69	<LOD	40.91 ± 0.33 ^b^	117.11 ± 0.87 ^a^	9.27 ± 0.84 ^c^	<LOD
8	Pelargonidin 3-O-glucoside	433.1131	433.1129	−0.52	<LOD	<LOD	<LOD	<LOD	528.55 ± 0.45 ^a^
9	Cyanidin 3-O-rutinoside	595.1666	595.1657	−1.46	758.12 ± 0.55 ^a^	35.43 ± 0.58 ^c^	110.38 ± 0.69 ^b^	12.5 ± 0.31 ^d^	<LOD
10	Pelargonidin 3,5-O-diglucoside	595.1662	595.1657	−1.38	136.39 ± 0.21^b^	<LOD	8.50 ± 0.20 ^c^	<LOD	778.84 ± 0.35 ^a^
11	Peonidin 3,5-O-diglucoside	625.1777	625.1763	−1.15	46.05 ± 0.54 ^b^	43.17 ± 0.29 ^b^	<LOD	110.38 ± 0.82 ^a^	<LOD
12	Pelargonidin 3-O-rutinoside	579.1722	579.1708	−2.41	<LOD	<LOD	<LOD	<LOD	206.87 ± 0.38 ^a^
13	Peonidin 3-O-rutinoside	609.1814	609.1814	0	<LOD	8.07 ± 0.32 ^c^	51.34 ± 0.38 ^a^	10.14 ± 0.41 ^bc^	19.42 ± 0.30 ^b^
14	Malvidin 3-O-rutinoside	639.1922	639.1920	−0.42	<LOD	32.54 ± 0.49 ^c^	135.72 ± 0.81 ^a^	60.76 ± 0.59 ^b^	<LOD

(Exp.): *m*/*z* experimental; (Calc.) *m*/*z* calculated by the Agilent Mass Hunter software (version B.07.00). * Compounds verified by a comparison with authentic chemical standards; LOD, limit of detection. ** The quantification of anthocyanins in all extracts was based on the calibration curves of the cyanidin 3-O-glucoside equivalent for monoglucosides and cyanidin 3,5-O-diglucoside for diglucosides. For each compound, the same letters indicate non-significant differences (*p* < 0.5) among different species.

**Table 2:** Quantification parameters of HPLC/MS analysis.

Compound	Equation of Curve	R^2^	Linear Range μg/ml	LOD μg/ml	LOQ μg/ml	RSD% Conc. Intra-Day (n = 5)	RSD% Conc. Inter-Day (n = 5)
Cy3gluc	Y = 377,129x – 94,124	0.999	1–80	0.3	1	0.21%	0.85%
Cy3,5digluc	Y = 381,475x – 85,623	0.998	1–80	0.3	1	0.23%	0.93%

LOD, limit of detection; LOQ limit of quantification; RSD% relative standard deviation; Cy3gluc, cyanidin 3-O-glucoside; Cy3,5 digluc, Cyanidin 3,5-O-diglucoside.

## Data Availability

The data presented in this study are available in the main article and in the Supplementary Materials.

## Supplementary Materials

The following supporting information can be downloaded at: https://www.mdpi.com/article/10.3390/plants14071050/s1, Figure S1: Analysis of conserved structural domains of Passiflora F3’H using BLASTP; Table S1: Primer utilized for qPCR analysis.
