# Supplementary material for: Anthocyanins and Anthocyanin Biosynthesis Gene Expression in Passiflora Flower Corona Filaments
Source: Plants (Basel). 2025 Mar 28;14(7):1050. doi: 10.3390/plants14071050 (PMC11991006; doi:10.3390/plants14071050)
Supplement: Supplementary file 1 [file plants-14-01050-s001.zip › plants-3531654-supplementary.pdf]

Supplementary Materials

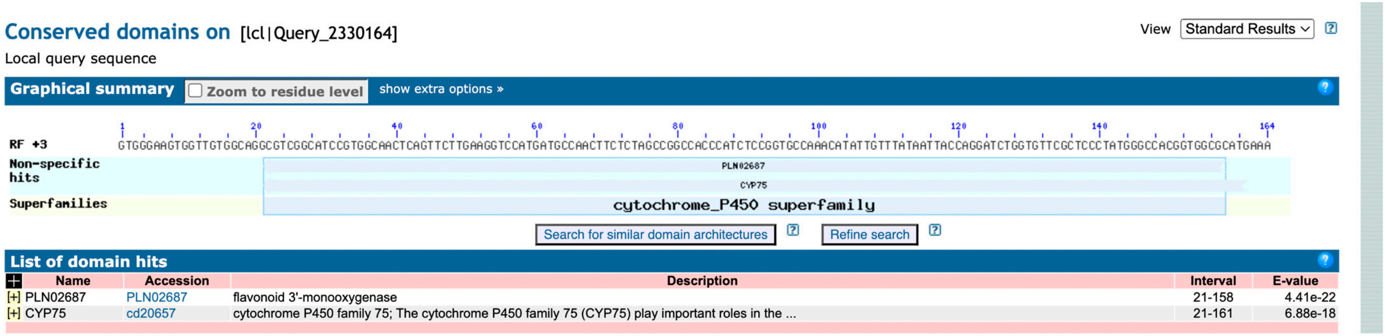

Figure S1. Analysis of conserved structural domains of *Passiflora* F3′H using BLASTP (https://blast.ncbi.nlm.nih.gov/Blast.cgi?PROGRAM=blastp&PAGE_TYPE=BlastSearch&LINK_LOC=blasthome).

Table S1. Primer utilized for qPCR analysis.

Gene	Gene full name	Forward primer (5′-3′)	Reverse primer (5′-3′)	Size (bp)	Source
DFR	*Dehydroflavonol reductase*	aagcgatacccatctgaccc	ccatccacggtcggtttaat	159	[36]
F3′H	*Flavonoid 3′- hydroxylase*	tgggaagtggttgtggcagg	tagggagcgaacaccagatc	141	This work
F3′5′H	*Flavonoid 3′ 5′-hydroxylase*	acgtcgtcaagcacagtaga	ggcttggaagtaggtgaggt	150	[37]
bHLH	*Basic helix-loop-helix (PebHLH126)*	tggtcttacgtcatgccagt	tgctattacactcgggcgat	151	[63]
MYB	*Myeloblastosis*	atctgaaacgcggcaacttt	cagtttgcggcgaatatggg	158	[36]
WD40	*WD40 protein*	aaattcgcccggatagcaac	ccacatggctgctatcatcg	156	[36]
EF 1-a	*Elongation factor 1-alpha*	ggcccaactggtctgactac	ttgcgggatcatccttggag	163	[37]
